# Molecular-Targeted Therapies for Hematologic Malignancies

**DOI:** 10.1155/2012/606423

**Published:** 2011-12-28

**Authors:** Kevin D. Bunting, Cheng-Kui Qu, Michael H. Tomasson

**Affiliations:** ^1^Aflac Cancer Center of Children's Healthcare of Atlanta and Emory University Department of Pediatrics, Atlanta, GA 30322, USA; ^2^Department of Medicine, Case Western Reserve University, Cleveland, OH 44106, USA; ^3^Division of Oncology, Department of Medicine, Washington University School of Medicine, St. Louis, MO 63110, USA

Major advances in the disciplines of hematology, genetics, biochemistry, and chemistry over the past decades have empowered investigators with the background and methods required for development of customized molecular-targeted therapies. The ability to identify signaling pathways that are dysregulated, to determine the associated mutations, and to develop chemical drugs toward a desired correction is now a realistic work flow. The landmark demonstration that BCR-ABL could be molecularly targeted and could have a major impact upon disease progression really ignited the field of targeted therapies for hematologic malignancies. Through much hard work, we now know the key drivers of some hematologic malignancies, and depending on the particular disease, we have an arsenal of agents available to act at multiple nodal points. Some enabling technologies that have been a key for these advances include small molecule screening, high throughput whole genome sequencing, mouse models for cancer, and gene and microRNA expression array analyses. In this special issue, we present a collection of seven articles that contribute to our understanding of molecular targets and the development of approaches for their inhibition or rationale use of existing agents or their derivatives. These targets range from very early initiators of malignancy to molecules that are already advanced into clinical studies. 

The first paper in this issue by A. Fathi and T. Abdel-Wahab “*Mutations in epigenetic modifiers in myeloid malignancies and the prospect of novel epigenetic-targeted therapy*” addresses the series of mutations identified that alter DNA and/or histone lysine methylation. These early epigenetic changes predispose to leukemogenesis and are relevant in acute myeloid leukemia (AML) and myelodysplastic syndrome (MDS) patients. The second paper by J. Okabe-Kado et al. “*Extracellular NM23 protein as a therapeutic target for hematologic malignancies*” describes extracellular NM23-H1 protein and its relationship with altered signaling pathways and growth/survival in AML. The third paper by S. Verma et al. “*Gab adapter proteins as therapeutic targets for hematologic disease*” describes the Grb2-associated adapter proteins (Gabs) as potential therapeutic targets playing major roles in regulation of multiple signaling pathways. These first three papers describe potential new targets that require further validation and greater specificity, but could have significant impact on the initiation and progression of hematologic malignancy. 

The fourth paper by X. Liu et al. “*Molecular targets for the treatment of juvenile myelomonocytic leukemia*” describes the outstanding progress made in identifying the mutations associated with activation of the Ras pathway in juvenile myelomonocytic leukemia (JMML), several of which are already targets of drugs that are being tested such as Ras and SHP-2. This paper additionally points toward protein:protein interactions as potential therapeutic targets such as SHP-2/Gab2 in hematopoiesis. The fifth paper by P. Argyriou et al. “*The role of mTOR inhibitors for the treatment of B-cell lymphomas*” focuses on the downstream activation of the mTOR pathway and the development and testing of new rapalogues and ATP-competitive inhibitors for clinical use. The mTOR pathway is central to cell survival and metabolism and represents a common target for many types of cancers. The sixth paper by F. Tzifi et al. “*The role of Bcl2 family of apoptosis regulator proteins in acute and chronic leukemias*” describes the exciting new advances in understanding and targeting the Bcl-2 family of proteins and gives a comprehensive update on new agents that are in clinical studies targeting survival in acute and chronic leukemias. All of these three papers describe *bona fide* targets that are already subject to significant validation and commercial drug development. 

The seventh paper by P. Koehler et al. “*Engineered T cells for the adoptive therapy of B cell-chronic lymphocytic leukemia (B-CLL)*” in the issue addresses immunotherapy using T-cell therapies against CD19, which has recently been very successful and received widespread attention for treatment of chronic lymphocytic leukemia. This approach promises to provide sustained targeted therapy based on cell surface phenotype and although it has to deal with issues such as B-cell deficiency and intravenous immunoglobuin infusions, such approaches when combined with chemotherapy are very promising as a form of targeted gene-based therapy.

In summary, the articles in this special issue address the spectrum of new targeted therapy development, from basic understandings of structure-function to mature rationale drugs already being tested in patients. We sought to cover the full spectrum of therapeutic development and are pleased to present a series of papers that do just that. As editors of this issue, we appreciate the important contributions of the authors of these review articles and hope that this issue will encourage expanded translational research toward developing novel therapies for hematologic malignancies. 

*Kevin D. Bunting* *Cheng-Kui Qu* *Michael H. Tomasson*

